# Peripubertal Testosterone, 17β-Estradiol and Progesterone Concentrations in Hair and Nails in Dobermann Dogs

**DOI:** 10.3390/ani13132241

**Published:** 2023-07-07

**Authors:** Jasmine Fusi, Maria Cristina Veronesi, Alberto Prandi, Monica Probo, Massimo Faustini, Tanja Peric

**Affiliations:** 1 Department of Veterinary Medicine and Animal Sciences, Università degli Studi di Milano, Via dell’Università 6, 26900 Lodi, Italy; jasmine.fusi@unimi.it (J.F.); maria.veronesi@unimi.it (M.C.V.); massimo.faustini@unimi.it (M.F.); 2 Department of Agricultural, Food, Environmental and Animal Sciences, University of Udine, Via Sondrio 2/a, 33100 Udine, Italy; alberto.prandi@uniud.it (A.P.); tanja.peric@uniud.it (T.P.)

**Keywords:** dog, puberty, hair, nails, testosterone, 17β-estradiol, progesterone

## Abstract

**Simple Summary:**

This study aimed to investigate the changes in sexual hormones around puberty in dogs by analyzing the concentrations of testosterone (T), 17β-estradiol (E2), and progesterone (P4) in hair and nails. Puberty in dogs is a complex process with various factors involved, making it challenging to study. Traditional methods using blood samples are not feasible for long-term studies. However, hair and nails have been identified as suitable biological specimens for retrospectively evaluating hormone accumulation. The study included five male and five female dogs. The results showed a significant increase in T levels in hair and nails of male dogs just before puberty. In females, there was a significant increase in E2 levels at puberty and a marked increase in P4 levels after puberty in both biological specimens. Sex-related differences were observed only in T concentrations in hair, but considering sex and sampling time together, T concentrations in hair and nails, as well as P4 concentrations in nails, were able to differentiate between male and female dogs at specific sampling times. This study demonstrates the usefulness of hair and nails as biological specimens for studying the changes in T, E2, and P4 during the peripubertal period in dogs.

**Abstract:**

Studies about puberty in dogs are few, probably because many factors are involved in the delicate process of puberty onset, leading to difficulties in the proper enrollment of subjects. Moreover, the use of blood for monitoring hormonal changes can be problematic, and not feasible for long-term studies. Hair and nails proved to be suitable matrices for the retrospective evaluation of hormones’ long-term accumulation. This study was performed using hair and nails for the evaluation of testosterone (T), 17β-estradiol (E2) and progesterone (P4) concentrations to assess possible sexual steroid changes during the peripubertal period in dogs. The results, obtained on five males and five females, showed a significant increase in T in hair and nails of males immediately before puberty. In females, a significant increase in E2 at puberty and a marked increase in P4 after puberty was found in both biological specimens. Sex-related differences were detected only for T hair concentrations, but when the sex and sampling time were considered together, hair and nails T and nails P4 concentrations allowed us to discern between male and female dogs at specific sampling times. The results from this study showed that hair and nails are useful biological specimens for the retrospective evaluation of changes in T, E2 and P4 concentrations in peripubertal dogs.

## 1. Introduction

In mammals, the onset of puberty, which signifies the beginning of reproduction, is influenced by a complex interplay of endocrinological and metabolic factors [1,2,3,4]. During this period, various interactions occur among factors such as the external environment, nervous system, and endocrine system. In females, puberty is marked by the initiation of ovarian activity, including the production of gametes and ovarian hormones, and the onset of the first estrus [5]. In males, puberty is characterized by the start of spermatogenesis, the first ejaculation [5,6], or the display of typical sexual behavior traits [4,7]. In female dogs (*Canis lupus familiaris*), puberty onset can be easily detected through the appearance of vaginal bleeding during the proestrus phase, the initial stage of the estrous cycle [7,8]. In common with other species, factors such as breed, genetics, body weight (BW), at puberty BW to adult BW ratio, environmental conditions, management, and socialization influence the achievement of puberty in dogs [4,9]. As a result of the complex interplay of these factors, sexual hormone levels, particularly testosterone (T) in males and estrogens (mainly 17β-estradiol, referred to as E2) and progesterone (P4) in females, rise around puberty [10].

Despite the importance of understanding hormonal changes during the crucial reproductive phase of puberty in dogs, there is a lack of scientific knowledge due to the long, gradual, and variable timeframe in achieving puberty [4]. In companion animals, the traditional use of blood for hormonal investigations poses challenges for studying this phase. Blood, although considered the conventional biological specimen for studying hormonal changes, is not ideal for long-term investigations, especially from an ethical standpoint, due to the need for frequent blood collections [11]. While blood sampling allows for the measurement of acute changes in hormone concentrations, it is not suitable for assessing the overall long-term changes in circulating hormones [12], which are highly characteristic of certain reproductive phases, such as the peripubertal period.

Hair has demonstrated its utility as a biological specimen for measuring long-term hormonal changes in humans and animals, as evidenced by numerous studies spanning over a decade [13]. The advantages of using hair include its non-invasive and easy collection, as well as the retrospective insight it provides into hormone accumulation over an extended period, thereby reducing the number and frequency of required samplings. Apart from hair, nails/claws have also been identified as a suitable biological specimen for hormone measurement in humans and animals, particularly for cortisol and/or dehydroepiandrosterone [14,15,16,17,18,19,20,21,22,23,24]. The affinity between lipophilic substances and nail keratin has been reported, indicating the ability of testosterone and estrogens to stably bind to keratin [18] and suggesting the usefulness of nails/claws in measuring sexual hormones [18]. Several studies [25,26,27,28,29] have utilized nails/claws to investigate sexual steroids in humans and animals. After an initial study demonstrated the usefulness of a single claws collection for hormone detection in deceased newborn puppies [17], subsequent studies showcased the feasibility of using claws for one-time or longitudinal hormone measurement in newborn puppies [22,28,30]. More recently, the suitability of repeated coat and claw collections for hormone measurements in female dogs, from the time of mating to puppies’ weaning, has been reported [31]. Thus, at present, coat and claws can be considered useful for measuring long-term hormone accumulation. However, to the best of our knowledge, no studies have investigated the potential changes in sexual steroids during the peripubertal period in dogs using the aforementioned biological specimens. Given the limited knowledge regarding sexual steroid changes around puberty in dogs, the present study aimed to measure testosterone (T), 17β-estradiol (E2), and progesterone (P4) concentrations in hair and nails around the time of puberty onset in dogs. Specifically, the study aimed to investigate: (1) potential time-related hormone changes in each matrix in males and females around puberty onset, and (2) possible hormonal differences between males and females.

## 2. Materials and Methods

### 2.1. Ethics

The study was conducted in accordance with EU Directive 2010/63/EU and approved by the Ethical Committee of the University of Milan (OPBA_147_2019). Written informed consent was obtained from the owners, allowing the collection of hair and nails samples and the recording of clinical data for research purposes.

### 2.2. Animals

The study was conducted between November 2021 and December 2022. Dogs were enrolled between November 2021 and February 2022 to ensure consistent climatic conditions and minimize seasonal changes. Only Dobermann dogs, belonging to a single dog breeder facility, recognized by the Fédération Cynologique Internationale (FCI) and located in Northern Italy, were included in the study. The facility had a humid-subtropical to a humid-continental and oceanic climate (Latitude 45°36′38″ N, Longitude 8°53′46″ E, at 222 m above sea level; temperature range: −8 °C to +32 °C). Each dog was housed in an outdoor kennel of 4 m^2^, which included an indoor sleeping area. Social interactions among puppies were limited to the same litter until 60 days of age, after which gradual socialization with puppies of the same age and young and adult dogs was provided, together with daily human handling. From weaning until the end of the study, all dogs were fed a specific dry diet for large breed requirements (large breed puppy, Hill’s Science Plan, Rome, Italy) and had free access to water throughout the day. Inclusion criteria for the study were: dogs of the same breed, bred in a single facility, puppies not sold by the breeder and retained for reproductive purposes, and provision of the same environmental, feeding, housing, handling, and social conditions. Exclusion criteria were: any disease or disturbance, even if transient, that could have interfered with the growth or wellbeing of the subjects. Other than this, every aspect not adherent with inclusion criteria caused the exclusion of the puppy. Puppies born from seven healthy bitches at term, with normal weight and no health issues from birth until the time of entering the study, were included. All dogs were monitored from 90 days of age until 2 months after puberty onset. Body weight (BW) was measured every 30 days using a digital weight scale platform (slim line platform scale, Eickemeyer Veterinary Equipment Ltd, Sunbury-on-Thames, Surrey, UK). The body condition score (BCS) was assessed on a 5-point scale (1 for emaciated, 5 for obese) [32] by the same investigator (JF) at each sampling time. Puberty onset in females was detected by observing proestral vaginal bleeding, which was confirmed by vaginal smears [4,33]. Vaginal smears were obtained by introducing a cotton-tipped swab into the vaginal canal, avoiding the clitoral fossa, and swabbing the dorsal vaginal wall. Cells from the swab were then transferred to a clean glass slide by gently rolling the swab. After Diff–Quik staining, smears were scanned on 40× and 200× to assess the percentage of exfoliated vaginal cells as the result of changes in circulating estrogens. [34]. Blood progesterone concentrations measured 20–40 days after heat by Minividas (Biomerieux, Grassina, Italia) were used to confirm diestrus in the five female dogs, considering blood P4 concentrations >10 ng/mL as indicative of diestrus [33]. In males, interest in females in heat was tested every 30 days. Puberty in males was considered after observing the typical rear leg lift during urination and/or obvious interest in females in heat, which was confirmed through successful semen collection and detection of spermatozoa [6,9]. Semen collection was performed by manual stimulation of the penis in presence of a bitch in estrus, using a latex artificial vagina attached to a glass, sterile, graduated tube, avoiding the complete collection of the third fraction. Immediately after collection, semen was assessed for volume, color, pH, and spermatozoa detection [35].

### 2.3. Hair and Nails Collection

To be sure of starting the study before puberty onset, hair and nail samplings were started at 90 days of age. Hair was collected by shaving an area of approximately 5 cm^2^ from the dorsal surface of the forearm to obtain enough regrown hair for subsequent samplings. Shaving was performed using a razor (TN2300 Nomad, Rowenta spa, Milan, Italy) until reaching the skin level. Once collected, hair was immediately placed in individually coded paper envelopes and stored in the dark at room temperature until analysis. The nail tips from all the forearm digits were clipped and pooled together. The nails were also placed in individually coded paper envelopes and stored at room temperature until analysis. Hair and nails were collected with gentle handling, without restraining the dogs. After each collection from a single dog, the razor and nail clipper were disinfected with a 70% alcohol solution [36]. To ensure a sufficient amount of biological specimen, regrown hair and nails were collected at fixed intervals of 30 days [31] until two samples after the puberty onset. The regrown part of the nail was identifiable based on its size, shape, and color, as previously reported in puppies [22,28,31]. Samples collected before puberty onset were considered prepubertal, while those obtained after puberty onset were considered postpubertal.

### 2.4. Hormonal Analysis

Hair and nails strands were washed in 3-mL isopropanol to ensure the removal of any steroids on their surface. Hair and nails steroids were extracted with 3-mL of methanol and measured using a solid-phase microtiter RIA assay, as described before [22,25,28,31]. The concentrations of progesterone, 17β-estradiol and testosterone were measured using a solid-phase microtiter RIA. In brief, a 96-well microtiter plate (OptiPlate; PerkinElmer Life Sciences Inc., Boston, MA, USA) was coated with goat anti-rabbit γ-globulin serum diluted 1:1000 in 0.15 mM sodium acetate buffer (pH 9) and incubated overnight at 4 °C. The plate was then washed twice with RIA buffer (pH 7.5) and incubated overnight at 4 °C with 200 μL of the antibody serum diluted 1:8000 for progesterone (Progesterone-11α-hemisuccinate-BSA; Lot RA/13; Analytical Antibodies, Bologna, Italy), 1:80,000 for 17β-estradiol (E2-6(O-carboxymethyl)-oxime-BSA; Lot 1; University of Udine, Udine, Italy), and 1:160,000 for testosterone (testosterone-3(O-carboxymethyl)-oxime-BSA; Lot RA/86/111; Analytical Antibodies, Bologna, Italy). The cross-reactivities of the anti-progesterone antibody with other steroids were as follows: progesterone, 100%, 11 β-OH-progesterone, 46%; 17α-OH-progesterone, 0.4%; 20α-OH-progesterone, 0.04%; testosterone, 0.08%; cortisol, <0.01%; 17β-estradiol, <0.01%; 17α-estradiol, <0.01%; and estrone, <0.01%. The cross-reactivities of the anti-17β-estradiol antibody with other steroids were as follows: 17β-estradiol, 100%; estrone, 2.5%; estriol, 0.12%; 17β-estradiol-(B-D-glucuronide), 0.04%; 17β-estradiol-3-sulfate 0.012%; DHEA, 0.007%; 17α-estradiol, <0.04%; progesterone, <0.04%; testosterone, <0.04%; androstenedione, <0.04%; estrone-3-sulfate, <0.04%. The cross-reactivities of the anti-testosterone antibody with other steroids were as follows: testosterone, 100%; 5α-dihydrotestosterone, 43.2%; 5α-androstanedione, 33.1%; 5β-androstanedione, 11.4%; 5α-androstan-3α,17β-diol, 9.4%; androstenedione, 0.4%; testosterone 17β-glucuronide, 0.09%; progesterone, DHEA, 17β-estradiol, androsterone-3-glucuronide, 0.01%; androsterone-3-glucuronide, 0.006%; cortisol, <0.001%. After washing the plate with RIA buffer, the standards (5–200 pg/well), the test extracts, and the tracer (progesterone [1,2,6,7-3H (N)];17β-estradiol [2,4,6,7-16-17-3H (N)] and testosterone [1,2,6,7-3H (N)] were added, and the plate was incubated overnight at 4 °C. Additionally, two quality controls from extracted samples were added to the plate: one had a low hormone concentration and the other had a high hormone concentration. The bound hormone was separated from the free hormone by decanting and washing the wells in RIA buffer. After the addition of 200 μL of a scintillation cocktail, the plate was counted on a β-counter (Top-Count; PerkinElmer Life Sciences Inc., Boston, MA, USA). The precision of the assay was estimated by assessing the intra- and inter-assay variation, which was expressed as the coefficient of variation (CV). The intra-assay precision was evaluated by testing 20 replicates of a sample with a known medium hormone concentration in the same assay. The inter-assay precision was evaluated by running the same sample in 20 assays in duplicate. The intra- and inter-assay CV were 3.4 and 8.2%, 4.1 and 11.1%, 4.2 and 12.3% for progesterone, 17β-estradiol and testosterone, respectively. The analytical sensitivity of the assay was determined as the hormone concentration that resulted in the displacement of the labeled hormone by at least 2 SD from the maximal binding (as calculated by RiaSmart; Canberra-Packard, Schwadorf, Austria). The sensitivities of the assays were 11.2 pg/mL, 15.4 pg/mL and 6.6 pg/mL for progesterone, 17β-estradiol and testosterone, respectively.

### 2.5. Statistical Analysis

To detect possible peripubertal hormonal changes in both biological specimens within each sex, data were submitted to ANOVA for repeated measures analysis, followed by the post hoc Holm test for multiple comparisons. The same test was also used to assess possible differences between the two sexes, considering the sampling time, the sex and the interaction between the two factors. Significance was set for *p* < 0.05. Data analysis was performed with the software JASP 0.17.1 for Windows, University of Amsterdam, The Netherlands.

## 3. Results

According to the previously described inclusion/exclusion criteria and to the minimum sample size indicated by the G*power test (ver. 3.1.9.6, Kiel University, Germany), 12 dogs were initially enrolled in the study. However, one dog was excluded as it was sold by the breeder at the age of 5 months. Additionally, one dog was excluded due to a severe gastroenteritis episode that occurred during the study and lasted approximately two weeks, negatively affecting its health and growth. Consequently, the study was conducted on a total of 10 Dobermann dogs, including 5 males and 5 females, all of which exhibited normal growth and optimal health throughout the study (Table S1). Among the enrolled dogs, two females and one male were littermates, while two other males were also littermates. The remaining five were unrelated to each other.

### 3.1. Puberty Onset

All five male dogs reached puberty between 9 and 12 months of age, while the five females reached puberty at 8–13 months of age. Puberty onset was confirmed through semen collection in males and vaginal smears in females: based on these examinations, the age at puberty was recorded for each subject. Blood progesterone analysis in females further confirmed the occurrence of diestrus in all individuals.

Table 1 presents data regarding sex, age, season (month) and daylight (hours), body weight (BW) in kilograms, body weight at puberty relative to adult body weight (%), and body condition score (BCS) at puberty onset for the 10 dogs.

**Figure 1:**
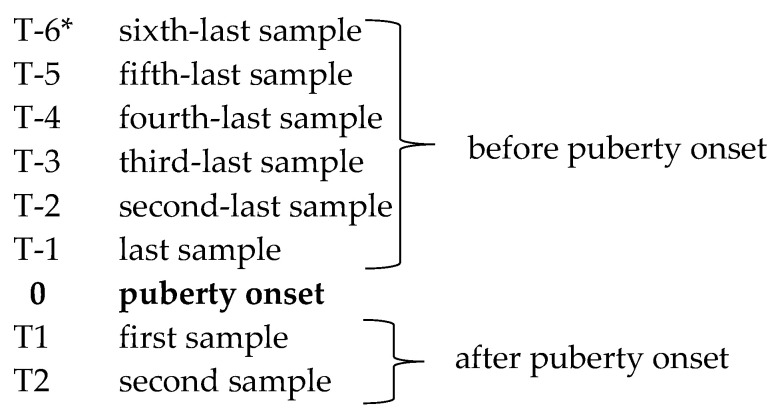
Sampling schedule. * performed only in males.

Given that the dogs reached puberty at different ages, we considered six prepubertal samplings from males and five from females for the analysis of hormonal changes during the prepubertal period. During the postpubertal period, all dogs underwent two sample collections. Thus, in males, the sampling times ranged from T-6 to T2 (total number of samples: eight per subject), while in females, they ranged from T-5 to T2 (total number of samples: seven per subject), as illustrated in Figure 1. Notably, none of the enrolled dogs experienced puberty onset simultaneously with the sampling times. It should also be mentioned that because puberty onset is a spontaneous event, the first postpubertal sampling (T1) occurred between 7 and 20 days after puberty onset, and consequently, the second postpubertal sampling (T2) was performed between 37 and 50 days after puberty onset.

### 3.2. Peripubertal Hormonal Changes

#### 3.2.1. Time-Related Hormonal Changes in Each Biological Specimen in Males

**Figure 2:**
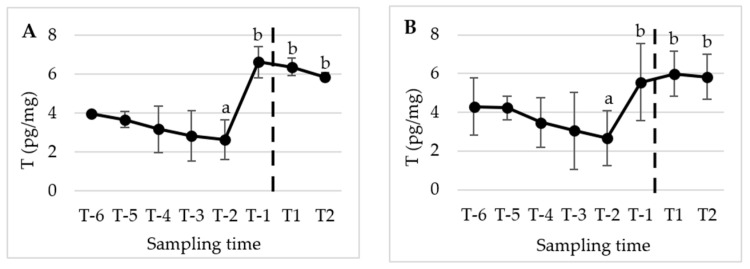
Mean (±SD) peripubertal (from T-6 to T2) hair (**A**) and nails (**B**) T concentrations in the 5 male dogs enrolled in the present study. (**A**) Dotted bar indicates puberty onset; a,b denote significant difference with *p* < 0.01. (**B**) Dotted bar indicates puberty onset; a,b denote significant difference with *p* < 0.05.

**Figure 3:**
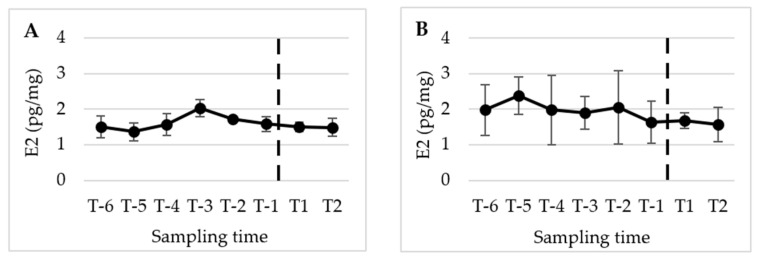
Mean (±SD) peripubertal (from T-6 to T2) hair (**A**) and nails (**B**) E2 concentrations in the 5 male dogs enrolled in the present study. (**A**,**B**) Dotted bar indicates puberty onset.

**Figure 4:**
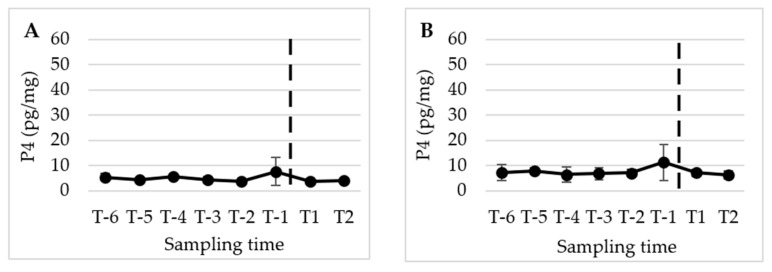
Mean (±SD) peripubertal (from T-6 to T2) hair (**A**) and nails (**B**) P4 concentrations in the 5 male dogs enrolled in the present study. (**A**,**B**) Dotted bar indicates puberty onset.

Figure 2, Figure 3 and Figure 4 present the mean (±SD) peripubertal (from T-6 to T2) concentrations of testosterone (T), 17β-estradiol (E2), and progesterone (P4) in hair and nails for the five male dogs included in this study.

The statistical analysis revealed a significant increase in T concentrations from T-2 to the subsequent sampling times, namely T-1, T1, and T2 (*p* < 0.01) in hair. However, no significant changes were observed in E2 and P4 levels over the course of the study. A similar pattern and timing were observed in the nails, with significant changes detected only for T (*p* < 0.05).

#### 3.2.2. Time-Related Hormonal Changes in Each Biological Specimen in Females

**Figure 5:**
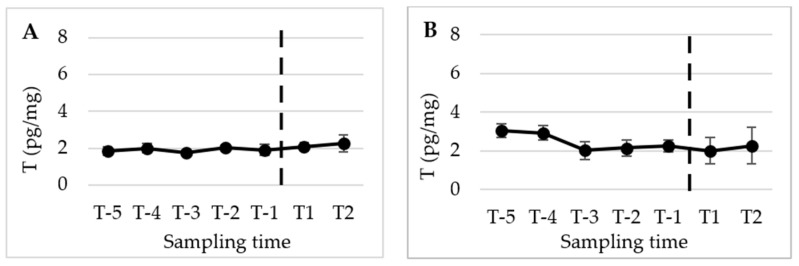
Mean (±SD) peripubertal (from T-5 to T2) hair (**A**) and nails (**B**) T concentrations in the 5 female dogs enrolled in the present study.

**Figure 6:**
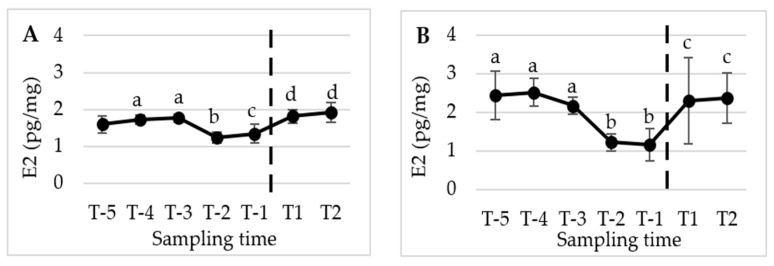
Mean (±SD) peripubertal (from T-5 to T2) hair (**A**) and nails (**B**) E2 concentrations in the 5 female dogs enrolled in the present study. (**A**) Dotted bar indicates puberty onset; a,b denote significant difference with *p* < 0.001; a,c denote significant difference with *p* < 0.05; b,d denote significant difference with *p* < 0.001; c,d denote significant difference with *p* < 0.05. (**B**) Dotted bar indicates puberty onset; a,b denote significant difference with *p* < 0.05; a,c denote significant difference with *p* < 0.05.

**Figure 7:**
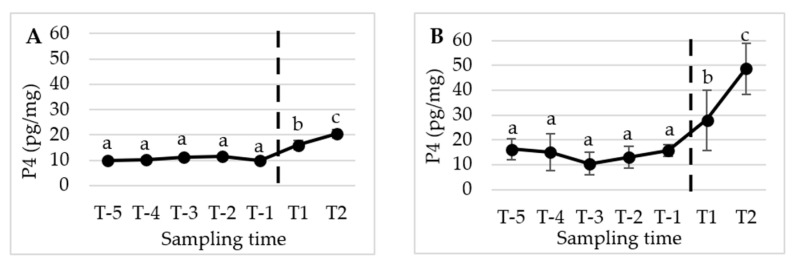
Mean (±SD) peripubertal (from T-5 to T2) hair (**A**) and nails (**B**) P4 concentrations in the 5 female dogs enrolled in the present study. (**A**) Dotted bar indicates puberty onset; a,b denote significant difference with p < 0.05; a,c denote significant difference with *p* < 0.01; b,c denote significant difference with *p* < 0.05. (**B**) Dotted bar indicates puberty onset; a,b denote significant difference with *p* < 0.01; a,c denote significant difference with *p* < 0.001; b,c denote significant difference with *p* < 0.01.

Figure 5, Figure 6 and Figure 7 display the mean (±SD) peripubertal (from T-5 to T2) concentrations of T, E2, and P4 in the hair and nails for the five female dogs included in the study.

In females, no significant time-related changes in T concentrations were observed in either the hair and nails (Figure 5). However, E2 concentrations showed a significant initial decrease in hair from T-4 and T-3 to T-2 (*p* < 0.001), and from T-4 and T-3 to T-1 (*p* < 0.05). Subsequently, a significant increase was observed between T-2 and T1, as well as between T-1 and T2 (*p* < 0.001 and *p* < 0.05, respectively). In the nails, E2 concentrations exhibited a similar pattern and timing to what was observed in the hair. Specifically, E2 nail concentrations significantly declined from T-5 and T-3 to T-2 and T-1 (*p* < 0.05), followed by an increase from T-2 and T-1 to T1 and T2 (*p* < 0.05) (Figure 6).

Regarding P4 concentrations, significant increases were observed in the hair between the sampling time interval T-5/T-1 compared to T1 (*p* < 0.05) and T2 (*p* < 0.01). Additionally, a significant increase was noted between T1 and T2 (*p* < 0.05). Progesterone concentrations in the nails displayed an identical pattern and timing of changes as observed in the hair, with a significant increase between the T-5/T-1 interval and T1 (*p* < 0.01) and T2 (*p* < 0.001). Similarly, a significant increase was observed between T1 and T2 (*p* < 0.01) (Figure 7).

#### 3.2.3. Sex-Related Differences

The statistical analysis demonstrated a significant influence of sex (*p* < 0.05) on T concentration in hair, with higher concentrations observed in males. An interaction between sex and sampling time was found at T-5 and T-1 (*p* < 0.001), indicating higher T concentrations in males. In nails, a significant interaction between sex and sampling time was detected at T-1 (*p* < 0.001), with higher T concentrations in males. Additionally, a significant interaction between sex and sampling time was found for P4 in nails at T2 (*p* < 0.001), with higher concentrations observed in females.

## 4. Discussion

The present study aimed to address the limitations associated with blood measurements in long-term hormonal studies by utilizing long-term retrospective biological specimens, specifically hair and nails, for hormone measurements during the peripubertal period in dogs. To the best of our knowledge, this study is the first to employ these biological specimens for hormonal analysis in dogs, confirming their usefulness [22,28,31].

The results obtained from the 10 enrolled Dobermann dogs within consistent breeding management revealed a wide range of puberty onset times, with greater inter-individual variability observed in females compared to males. Despite the individual variations, the average age at puberty onset did not differ significantly between genders, with females reaching puberty at around 11 months and males at approximately 10.4 months. This finding differs from previous reports indicating earlier puberty onset in males compared to females [9]. Evaluating the influence of body weight and percentage of BW on puberty onset, our results showed that all subjects had a percentage of BW relative to adult BW consistently above 70–72%, even though three dogs had percentages lower than the suggested threshold of 81% of mature BW for the onset of puberty [4], based on previous studies [37,38]. Based on the body condition score (BCS) at puberty onset, all dogs exhibited optimal body conditions with BCSs ranging between 2.5/5 and 3/5. Therefore, these findings suggest that sufficient body development was attained at the time of puberty onset in the enrolled dogs. Regarding the season in which puberty occurred, four dogs (two males and two females) reached puberty in spring, two males in summer, and four dogs (three females and one male) in autumn. However, due to the limited number of animals enrolled, a statistical analysis of the combined effects of age, BW, and season on puberty occurrence was not possible, which is a limitation of this study. Nevertheless, although dogs are generally considered non-seasonal from a reproductive perspective [9], it would be interesting to investigate whether age, BW, season (and daylight), or their interactions influence puberty onset in dogs. Descriptively, it is noteworthy that littermate males attained puberty at the same age and with similar BW, and a similar pattern was observed among the other littermates, where the two females reached puberty at the same age, closely matching the age at which the male littermate attained puberty. Together with family lines and breed, it is possible to suppose that genetics is one of the factors driving the timing of puberty onset, as observed in this study [7,9]. However, to draw conclusions on this point more animals should be enrolled.

Focusing on peripubertal hormone changes in male dogs, our results showed a significant approximately twofold increase in testosterone (T) accumulation in both biological specimens immediately before puberty, which remained high until the end of the study, indicating that T secretion by the testes precedes the onset of puberty. The average >2.6 pg/mg concentrations of T in the hair were higher than the range of 0.3–2.7 pg/mg reported in men’s hair [27], and the average T concentrations in nails were threefold higher than the 0.4–0.8 pg/mg reported in human nails [39]. It should be noted, however, that different laboratory techniques were employed in those studies compared to our study, potentially accounting for the divergent concentrations found. Nevertheless, the similar profiles of T concentrations in both hair and nails suggest that both biological specimens can serve as alternatives to assess changes in T concentration in dogs. The concentrations of E2 and P4 in males did not exhibit significant changes over the study period in either biological specimen, with similar profiles and low concentrations, albeit about tenfold higher than the P4 concentrations reported in nails in men (1.0–2.1 pg/mg) [39].

In females, the statistical analysis revealed a biphasic pattern of E2 concentration changes, which were quite similar in hair and nails, although E2 concentrations in nails were about 70% higher. A decline in E2 concentrations was observed approximately one to two samplings prior to puberty achievement, followed by a significant approximately 70% increase in hair, and about 50% increase in nails at T1 and T2. This finding could be attributed to follicular development occurring before puberty, leading to increased estrogen secretion and circulation [9], surpassing the threshold required to trigger GnRH secretion by the surge center of the hypothalamus [9]. However, the precise mechanisms by which estradiol controls GnRH secretion in the hypothalamus during the peripubertal phase remain largely unknown, as stated by Senger [9]. With regard to P4 concentrations, although the profiles were similar between the two biological specimens, the average P4 concentrations were about twofold higher in nails than in hair. Both profiles exhibited an approximately twofold increase in P4 concentrations at the first and even more so at the second sampling after puberty, reflecting the onset of the diestrus phase in all bitches and confirming ovulation in all subjects, as detected by plasma progesterone evaluation. It is not surprising that the highest P4 concentrations were observed at the second postpubertal sampling time, as both biological specimens had been exposed to accumulating circulating hormones for a longer duration. Compared to P4 concentrations in claws from calves and pregnant cows [25], P4 concentrations in nails in our study were very similar to the ~20 pg/mg reported for calves and fell within the wide range of 14.32–103.66 pg/mg reported for pregnant cows, but were more than twofold higher than the range of 0.7–12.8 pg/mg reported in women using a different laboratory technique [39]. Because the laboratory technique employed by Comin et al. [25] was similar to ours, it can be assumed that, although species-related differences must be considered, sexual steroid concentrations in nails/claws are strongly influenced by the method of analysis. Testosterone profiles did not exhibit significant changes in both biological specimens, indicating that, although present in females as well, as reported in women [27], T does not play a significant role in peripubertal hormonal changes in female dogs.

Regarding the differences between the sexes, the statistical analysis demonstrated that considering the interaction between sex and sampling time provides a better estimation of hormone differences in longitudinal studies. A clear sex effect was observed only in T concentration in hair, whereas for T and P4 in nails, the sex-related differences were influenced by the interaction between sex and sampling time. Therefore, based on the present results, a definitive sex effect can only be considered for T in hair. The higher T concentrations in the hair of males align with the study by Fusi et al. [29] in adult cats, where only T assessed in the coat from a single sampling time proved useful in distinguishing between postpubertal males and females. Furthermore, a previous study in humans [27] demonstrated that gender influences hair concentrations of T and P4, with a tendency towards higher T in the hair of males and higher P4 in the hair of females.

Furthermore, a comment on hair hormonal concentrations concerns the possible influence of breed-specific hair characteristics on hormone accumulations. In fact, although the authors are not aware of studies investigating the possible influence of different breed-related types of hair in dogs on the ability to incorporate hormones, this aspect should not be excluded and requires further focused investigation. A last comment regards a strength of the study. Puberty studies in dogs are scarce due to the complex nature of this process, making the selection and enrolment of appropriate animals challenging [4]. In this study, animal selection was very strict to minimize the influence of various factors. For this reason, the number of subjects was limited to five males and five females. Animals of a single dog breed, belonging to a single breeder, and subjected to the same housing, handling, nutrition, and socialization conditions were included to mitigate the potential impacts of breed, breeding system, environment, and management, as suggested by Gobello [4]. Additionally, the timing of the initial recruitment was restricted to minimize the influence of climate conditions and the photoperiod on growth and development.

## 5. Conclusions

In summary, the findings of this study confirm the usefulness of both hair and nails as biological specimens for investigating the retrospective changes in T, E2 and P4 concentrations during the peripubertal period in dogs. In male dogs, T concentrations increased prior to puberty onset and remained elevated thereafter, indicating the role of testicular secretion in driving T accumulation. In females, a significant increase in E2 concentrations at puberty was observed in both hair and nails, likely attributed to heightened estrogen secretion and circulation associated with follicular development before puberty. Moreover, a marked increase in P4 concentrations following puberty was detected in both biological specimens, reflecting the attainment of the diestrus phase. While sex-related differences were observed only in T concentrations in the hair, considering the interaction between the sex and sampling time yielded a better ability to distinguish between male and female dogs. Collectively, these findings highlight the value of hair and nails as valuable biological specimens for studying the complex peripubertal reproductive stage in dogs. However, further investigations are warranted to explore the mechanisms underlying sexual hormone accumulation in these distinct biological matrices and their implications.

## Figures and Tables

**Table 1:** Data about gender, age, season (month) and daylight (h), BW (kg), BW at puberty with respect to adult BW (%), and BCS at puberty onset of the 10 dogs enrolled in the present study.

Dogs	Gender	Age at Puberty (Months)	Season (Month) at Puberty and Mean Daylight (h)	BW at Puberty (kg)	BW at Puberty with Respect to Adult BW ^#^ (%)	BCS at Puberty
1	female	8	Spring (April) (13 h 40 min)	27	77	2.5
2 *	female	13	Autumn (November) (9 h 30 min)	35	100	3
3	female	8	Spring (May) (15 h 20 min)	29	83	2.5
4	female	13	Autumn (November) (9 h 30 min)	34	97	3
5 *	female	13	Autumn (November) (9 h 30 min)	32	91	3
6	male	11	Summer (September) (12 h 30 min)	39	87	2.5
7	male	11	Summer (July) (15 h 40 min)	40	89	3
8 *	male	12	Autumn (October) (11 h 20 min)	39	87	2.5
9 ^§^	male	9	Spring (May) (15 h 20 min)	33	73	3
10 ^§^	male	9	Spring (May) (15 h 20 min)	33	73	3

* Littermates; ^§^ littermates; ^#^ Dobermann adult BW: 30–35 kg in females; 40–45 kg in males (FCI, Fédération Cynologique Internationale).

## Data Availability

Data are available upon reasonable request to the corresponding author.

## Supplementary Materials

The following supporting information can be downloaded at: https://www.mdpi.com/article/10.3390/ani13132241/s1. Table S1: Body Weight (kg) from 3 to 15 months of age in the five male and five female Dobermann dogs enrolled in the study.
