# Supplementary material for: Peripubertal Testosterone, 17β-Estradiol and Progesterone Concentrations in Hair and Nails in Dobermann Dogs
Source: Animals (Basel). 2023 Jul 7;13(13):2241. doi: 10.3390/ani13132241 (PMC10339877; doi:10.3390/ani13132241)
Supplement: Supplementary file 1 [file animals-13-02241-s001.zip › animals-2460237-supplementary.pdf]

Table S1. Body Weight (kg) from 3 to 15 months of age in the 5 male and 5 female Dobermann dogs enrolled in the present study

dog	3 months	4	5	6	7	8	9	10	11	12	13	14	15
female 1	11	14	18	21	24	27	25	26	28	29	31	32	32
female 2*	11	15	18	20	23	25	26	28	29	30	32	33	34
female 3*	13	17	21	25	28	31	32	33	34	34	35	35	35
female 4	12	17	20	24	28	29	32	32	33	33	34	34	35
female 5	13	16	19	23	27	29	30	31	32	33	34	35	35
mean	12	15,8	19,2	22,6	26	28,2	29	30	31,2	31,8	33,2	33,8	34,2
sd	1,00	1,30	1,30	2,07	2,35	2,28	3,32	2,92	2,59	2,17	1,64	1,30	1,30
male 1*	13	17	21	24	26	29	33	35	37	39	41	43	44
male 2	15	19	23	27	29	32	35	37	39	42	43	44	45
male 3	15	19	23	28	30	33	36	38	40	43	45	45	45
male 4§	13	17	21	25	28	30	33	35	38	40	41	42	44
male 5§	14	18	21	25	27	29	33	35	38	41	42	43	44
mean	14	18	21,8	25,8	28	30,6	34	36	38,4	41	42,4	43,4	44,4
sd	1,00	1,00	1,10	1,64	1,58	1,82	1,41	1,41	1,14	1,58	1,67	1,14	0,55

* littermates
§ littermates
